# The Inflating Impact of Waiting-List Controls on Effect Size Estimates

**DOI:** 10.3389/fpsyt.2022.877089

**Published:** 2022-06-22

**Authors:** Keith R. Laws, Luca Pellegrini, Jemma E. Reid, Lynne M. Drummond, Naomi A. Fineberg

**Affiliations:** ^1^School of Life and Medical Sciences, University of Hertfordshire, Hatfield, United Kingdom; ^2^Hertfordshire Partnership University NHS Foundation Trust, Welwyn Garden City, United Kingdom; ^3^Centre for Psychedelic Research, Imperial College London, London, United Kingdom; ^4^South West London and St George's NHS Trust, London, United Kingdom; ^5^Hertfordshire Partnership University NHS Foundation Trust, Hatfield, United Kingdom; ^6^University of Cambridge School of Clinical Medicine, Cambridge, United Kingdom

**Keywords:** obsessive compulsive disorder (OCD), psychological therapies, waiting list control groups, meta-analysis, moderator analyses

## Introduction

The primary psychological intervention recommended for obsessive compulsive disorder (OCD) is cognitive behavioral therapy (CBT) with exposure and response prevention (ERP), in which individuals are taught to confront and tolerate conditions that provoke obsessions and compulsions and resist acting on them. A recently published systematic review and meta-analysis of 24 studies by Ferrando and Selai (1) reports a large mean effect size in favor of ERP for reducing OCD symptomatology [−0.75, (95% CI = −0.92 to −0.59)], with the authors endorsing ERP as “the treatment of choice for OCD.” (p. 10). We highlight below a series of methodological issues that contextualize and temper such strong advocacy for ERP.

The overall effect size of ERP for OCD reported by Ferrando and Selai (1) is similar to that reported earlier by us (2) (g = 0.74: 95% CI = 0.51 to 0.97, k = 36). Any similarity, however, may be misleading since Ferrando and Selai determined effect sizes using between-group pre-post change-scores and a fixed-effects model, while we analyzed end-of-trial between-group effect sizes using a random effects approach and on a 50% larger sample of randomized controlled trials (RCT). Another key difference between the two meta-analyses concerns the importance of addressing the impact of factors known to moderate the effect sizes of psychological interventions in this population. In particular, we argue that the conclusions of Ferrando and Selai are overly optimistic because they did not examine the moderating effect of type of control group, and its associated impact on the heterogeneity in their results –and that had they done so, this would have substantially altered the reported efficacy of ERP for OCD.

The choice of control group (e.g., a waiting list, another form of psychological treatment, or treatment as usual) has repeatedly been shown to moderate the effect size of psychological treatments for OCD [e.g., (2–4)]. Although Ferrando and Selai state that ERP was superior to various controls, they did not compare ERP outcomes across different control groups. This point is important to clarify, because their abstract states that “Our review suggests that ERP was superior to the other groups, including *both neutral and active treatments*, in reducing OCD symptomatology.” Similarly, they open their discussion with “Our main aim was to determine whether ERP-based therapy was more effective in reducing OCD symptoms *compared to no treatment or other psychotherapeutic or pharmaceutical interventions.”* Later in the discussion they similarly say “The efficacy of ERP therapy in reducing OCD symptoms was compared to a control group, *either waiting-list or placebo, or another therapy*, which included anxiety management, cognitive therapy, IBA, autogenic training, relaxation therapy, fluoxetine, sertraline and eye-movement desensitization and reprocessing” (our italics). These descriptions could be interpreted to mean that ERP was compared against each of these different controls, but their analysis only compares ERP with *all* control conditions collectively.

Using the data presented by Ferrando and Selai, we calculated effect sizes for ERP vs. waiting-list controls [−1.82 (−2.11 to −1.52; k = 8], which was significantly larger than the ERP comparisons vs. all remaining control conditions [−0.32 (−0.52 to −0.13; k = 15] (Q = 11.67, *p* < 0.001). Indeed, we found that whether the trials used waiting-list or other controls accounted for one third of the variance in the effect sizes [R^2^ analog = 0.33; and removal of one extreme outlier (5) increased the R^2^ analog = 0.41]. Thus, by pooling data across all control groups Ferrando and Selai do not address this important factor, with effect sizes for ERP being almost 6 times larger when compared to waiting-list rather than to other control conditions. Further, this re-analysis of their data accords with our own meta-analysis (2), which showed similar large effect sizes for ERP vs. waiting-list (g = 1.27: 95% CI 0.79–1.75, k = 8), but not when we compared to active psychological therapy controls (g= −0.05: 95% CI −0.27 to 0.16, k = 8).

Since wait-list comparisons comprised a substantial proportion (one-third) of all ERP trials included by Ferrando and Selai, it is not surprising that they report extremely high levels of heterogeneity. From their reported Q = 185.66, we estimate an I^2^ of 87%, which would represent a “severe” level of heterogeneity. Ferrando and Selai state that “Our test for heterogeneity was significant (Q-statistic = 185.66, *p* < *0.0*1), indicating that one study could be driving the results as there is some unexplained heterogeneity between studies. Cochrane guidelines, used throughout this piece, report that when using a fixed-effects model the heterogeneity result can be ignored (6).” However, Cochrane rightly state that fixed-effect meta-analyses ignore heterogeneity, not that heterogeneity can be ignored. Heterogeneity is extremely high and this is neither a reason for using a fixed-effects analysis nor for assuming that a fixed-effects analysis negates concerns about heterogeneity. We would question the selection of a fixed-effects rather than a random-effects model. It seems implausible that the included samples are derived from the same general population and estimating the same underlying fixed treatment effect. This is underscored by the extreme heterogeneity for individual study effect sizes reported by Ferrando and Selai, which ranges massively from + 1.03– −16.01.

Ferrando and Selai did conduct a leave-1-out analysis, where the effect size is recalculated after removing each individual study; and they report it always remained significant. In this context, they identified three trials (7–9) as outliers and ran the meta-analysis removing these three outliers. They report that their “…original findings were unaffected by the removal of these studies {effect size = −0.97, significant at *p* < *0.0*1 [95% CI = (−1.18 to −0.76); SE = 0.11, *z* = −8.94]}. This, combined with the leave 1 out test, demonstrates that our original results can be considered valid.” Although the leave-1-out test remains significant following each removal, it is important to examine the *range* of effect sizes that emerge. Our re-assessment of this analysis suggests that effect sizes vary considerably–between −0.64 (leaving out Gomes et al.) to −0.96 (leaving out Belloto-Silva et al.). In other words, these individual studies have a large impact on the reported effect size. Removal of these three studies increased the overall effect size by ~30% from −0.75 to −0.97 (−1.18 to −0.76). We note that the new lower 95% CI of −0.76 exceeds the mean across the original sample (−0.75). Finally, we note that these 3 studies undoubtedly influence the overall results having the largest samples and their weighting being greatest in the analysis. In fact, these 3 trials comprise over 30% of the participants across all 24 RCTs analyzed by Ferrando and Selai. In this context, it is worth noting that larger trials would be expected to produce the most accurate effect size estimates–and these three larger trials alone produce a much smaller pooled effect size (−0.41, 95% CI −0.68 to −0.15), which is less than half of the −0.97 estimated by Ferrando and Selai (after their removal).

The authors do not present funnel plots or check for possible publication bias; however, our re-analysis of their data (using their own fixed effects model) indicates some funnel plot asymmetry; and a trim and fill analysis points to 4 potentially missing studies that would reduce the effect size to −0.65 (95% CI −0.81 to −0.49). This possible publication bias is underscored by Begg and Mazumdar rank correlation, which is significant (z for tau 1.77, *p* = 0.03); and Egger's intercept −3.52 (*p* < 0.01) also pointing to possible presence of publication bias. Inspection of their forest plot also identifies evidence of extreme outliers. For example, Khodarahimi (5) has an effect size of −16.01 (95%CI −20.98 to −11.03); however, removal of this study does not reduce the I^2^ value (which is still extremely high at I2 = 86.67%). Clearly, multiple studies have produced unusual effect sizes. We also note that Simpson et al. appears to be missing from the overall effect size calculation (see forest plot).

To summarize, we argue that the effect size reported by Ferrando and Selai exaggerates the efficacy of ERP at reducing OCD symptoms. As with our own meta-analysis (2), our re-analysis of the data here shows that waiting list control groups inflate effect sizes [see (10, 11)]. As noted by Leichsenring and Steinert (12) “When examining efficacy, a treatment may be compared with different comparators, that is, with an established treatment, treatment as usual, a placebo, or a waiting list, with *decreasing strictness of the empirical test*.” (p. 1,323, our italics). Several factors might lead to the exaggerated difference for waiting-list controls. It may be that only some patients are willing to be randomized to waiting-list control conditions e.g., if they have particularly high expectations for the therapy (13)–such factors might both inflate the intervention and deflate the control. Thus, we question the conclusion that ERP should be considered the treatment of choice for OCD. Only comparisons with *no treatment* indicate large effects, while those with other types of control groups identify much more modest evidence for ERP efficacy.

## Author Contributions

KL and NF conceived of the initial study and created an initial draft. LP, LD, and JR commented on and contributed to the initial draft. All authors approve the current version of the paper.

## Conflict of Interest

In the last three years, NF has received publishing royalties from Oxford University Press and an honorarium from Elsevier for editorial work. In the past she has received funding from various pharmaceutical companies for research into the role of various medications as treatments for OCD, for giving lectures and webinars and for attending scientific meetings. The remaining authors declare that the research was conducted in the absence of any commercial or financial relationships that could be construed as a potential conflict of interest.

## Publisher's Note

All claims expressed in this article are solely those of the authors and do not necessarily represent those of their affiliated organizations, or those of the publisher, the editors and the reviewers. Any product that may be evaluated in this article, or claim that may be made by its manufacturer, is not guaranteed or endorsed by the publisher.
